# Influence of Diet and Levels of Zonulin, Lipopolysaccharide and C-Reactive Protein on Cardiometabolic Risk Factors in Young Subjects

**DOI:** 10.3390/nu13124472

**Published:** 2021-12-15

**Authors:** Constanza C. Astudillo-López, Natividad Castro-Alarcón, Ana C. Ariza, José F. Muñoz-Valle, Ulises de la Cruz-Mosso, Eugenia Flores-Alfaro, Oscar del Moral-Hernández, Ma. Elena Moreno-Godínez, Marco A. Ramírez-Vargas, Inés Matia-Garcia, Isela Parra-Rojas

**Affiliations:** 1 Facultad de Ciencias Químico Biológicas, Universidad Autónoma de Guerrero, Chilpancingo 39090, Guerrero, Mexico; cony-astudillo@hotmail.com (C.C.A.-L.); natycastro2@hotmail.com (N.C.-A.); efloresa_2@hotmail.com (E.F.-A.); odelmoralh@gmail.com (O.d.M.-H.); emoreno20@hotmail.com (M.E.M.-G.); marvar@uagro.mx (M.A.R.-V.); inesita-86@hotmail.com (I.M.-G.); 2 Consejo Nacional de Ciencia y Tecnología—Centro de Investigación en Nutrición y Salud, Instituto Nacional de Salud Pública, Cuernavaca 62100, Morelos, Mexico; carolina.ariza@insp.mx; 3 Instituto de Investigación en Ciencias Biomédicas, Centro Universitario de Ciencias de la Salud, Universidad de Guadalajara, Guadalajara 44340, Jalisco, Mexico; biologiamolecular@hotmail.com; 4 Instituto de Nutrigenética y Nutrigenómica Traslacional, Centro Universitario de Ciencias de la Salud, Universidad de Guadalajara, Guadalajara 44340, Jalisco, Mexico; ulises_cdm@hotmail.com

**Keywords:** zonulin, lipopolysaccharide, C-reactive protein, intestinal permeability, cardiometabolic risk

## Abstract

A western diet and increased intestinal permeability may contribute to systemic inflammation and the development of cardio-metabolic alterations. The aim of this study was to assess the relationship between diet, biomarkers of intestinal permeability, and chronic low-grade inflammation on the cardiometabolic profile. A cross-sectional study was carried out in 238 young subjects aged 18–29 years, divided into two groups: with <3 cardiometabolic risk factors (CRF) and ≥3 risk factors. Anthropometric parameters, biochemical profile, and serum levels of zonulin, lipopolysaccharide (LPS), and high-sensitivity C-reactive protein (hs-CRP) were measured, and the macronutrient intake was evaluated. Interaction models showed elevated glucose levels in the presence of high biomarker levels: zonulin ≥51.6 ng/mL plus LPS ≥ 1.35 EU/mL (β = 1.1, *p* = 0.006), and LPS ≥1.35 EU/mL plus hs-CRP ≥ 4.3 mg/L (β = 1.2, *p* = 0.007). In addition, triglyceride levels increased in the presence of LPS ≥ 1.35 EU/mL and hs-CRP ≥ 4.3 mg/L (β = 3.9, *p* = 0.01). Despite having increased biomarker levels, a higher consumption of water (≥2100 mL), polyunsaturated fatty acids (≥6.0 g), or fiber (≥30 g) decreased triglyceride (β = −9.6, *p* = 0.03), total cholesterol (β = −5.1, *p* = 0.01), and LDL-C levels (β = −7.7, *p* = 0.01). These findings suggest that the increased consumption of water, PUFA and fiber may improve lipid profile in subjects with intestinal permeability dysfunction or low-grade systemic inflammation.

## 1. Introduction

Non-communicable diseases, also known as chronic diseases, are caused by a combination of genetic, physiological, lifestyle and environmental factors. From 1990 to 2017, the absolute number of deaths from preventable non-communicable diseases increased 49.3% [1]. Particularly, changes in lifestyle patterns lead to the development of obesity, stroke, stress, atherosclerosis, cancer, diabetes, and other non-communicable diseases [2]. In Latin America and the Caribbean, the double burden of undernutrition is linked to the consumption of ultra-processed, energy-dense foods rich in fat, sugars, or salt and provide no nutrients which are a major cause of diseases associated with poor nutrition [3].

An unhealthy diet is one of the key modifiable risk factors in the development of metabolic alterations associated with non-communicable diseases, not only due to the total amount of macronutrients consumed but also to the type and amount of nutrients ingested [4]. There is evidence that low-carbohydrate diets can improve insulin sensitivity, whereas the high intake of carbohydrates with a high glycemic index can promote insulin resistance, either through loss of pancreatic function, excessive insulin secretion, or beta-cell glucose toxicity [5]. Lower intake of saturated fatty acids (SFA) and increased consumption of polyunsaturated fatty acids (PUFA) have also been shown to improve blood pressure, coagulation, endothelial function, and insulin resistance. Furthermore, in several cross-sectional and intervention studies, an increase in fiber intake has been associated with a decrease in insulin resistance, and thus with a lower prevalence of metabolic disorders [6].

In recent years, increased intestinal permeability has been suggested as a risk factor for systemic inflammation since it promotes a proinflammatory phenotype induced by increased paracellular translocation of microbial antigens across the intestinal barrier [7], and the subsequent increased release of lipopolysaccharide (LPS). LPS from Gram-negative bacteria is mobilized into the blood circulation and induces an acute or chronic inflammatory response due to the release of pro-inflammatory cytokines, mediators of inflammation, such as C-reactive protein, and immune cells activation [8].

One of the mechanisms that favor the entry of LPS into the blood circulation is a high-fat diet since chylomicrons transport LPS through the intestinal epithelium [9]. Another route of LPS entry is passive diffusion when there is structural damage of the pore formed by tight junction proteins, which prevent the entry of LPS into the blood circulation [10]. The zonulin protein derived from enterocytes regulates tight junction reorganization increasing its synthesis in response to specific stimuli, such as enteric bacteria and gliadin protein from wheat. Zonulin also increases intestinal permeability and facilitates the entry of LPS, which in turn produces chronic subclinical inflammation [11].

Several studies have found increased serum levels of zonulin, LPS, and high-sensitivity C-reactive protein (hs-CRP) in patients with coronary heart disease and type 2 diabetes, associated with increased waist circumference, insulin resistance, dyslipidemia, inflammation, diastolic blood pressure, and fasting glucose [12,13,14,15]. Therefore, the aim of the study was to analyze the relationship of diet with serum levels of zonulin, LPS and hs-CRP on cardiometabolic risk factors in young subjects. 

## 2. Materials and Methods

### 2.1. Participants

A cross-sectional study that included 238 young subjects aged between 18–29 years from the Autonomous University of Guerrero in Mexico, was carried out. Inclusion criteria were subjects without gastric, hepatic, renal, or thyroid disease, nor infectious diseases, and without pharmacological treatment. Pregnant women or women undergoing hormonal treatment were excluded. Participants signed an informed consent form according to the Declaration of Helsinki. The study was approved by the Research Ethics Committee of the Autonomous University of Guerrero (CB-002/2018).

### 2.2. Clinical Measurements

Anthropometric measurements were obtained in the morning after an 8 to 12 h fast. Body mass index (BMI) was determined with a body composition analyzer (MC-780U TANITA, Arlington Heights, Illinois, USA) that included the following data: weight (kg), BMI (kg/m^2^), fat mass (kg), and lean mass (kg). For these measurements, participants were asked to wear light clothing, without accessories, and be barefoot. An anthropometric tape (Seca 203, Hamburg, Germany) was used to measure body circumferences. Central obesity is an excess of body fat in the abdomen, determined when the waist circumference is ≥80 cm in women and ≥90 cm in men, according to the International Diabetes Federation [16]. Blood pressure was measured in duplicate with an automatic digital blood pressure monitor (HEM-7120 Omron Healthcare Inc., Illinois, IL, USA), which was determined as high blood pressure when found ≥130/85 mmHg [16]. All measurements were performed by trained personnel. 

### 2.3. Nutritional Assessment

The average intake of macronutrients was assessed by means of a multi-pass 24-h dietary recall, which was performed in the morning from Monday to Friday. This questionnaire collected food intake data from the previous day; the person responsible for applying the questionnaire was a nutritionist trained in the methodology [17]. The calculation of the energy and nutritional content was performed with Nutrimind 2013 software by entering the intake of each nutrient in grams obtained from the 24-h dietary recall [18].

A high intake of macronutrients was considered above the third tertile, total energy ≥ 3236 kcal, total fat ≥138 g, carbohydrates ≥ 406 g, proteins ≥ 133 g, cholesterol ≥ 445 mg, saturated fatty acids (SFA) ≥ 24.6 g, polyunsaturated fatty acids (PUFA) ≥ 6 g, dietary fiber ≥ 30 g, and water ≥ 2100 mL.

### 2.4. Biochemical Measurements and Definitions

Serum concentrations of glucose, triglycerides, total cholesterol, low-density lipoprotein cholesterol (LDL-C), and high-density lipoprotein cholesterol (HDL-C) were measured with a clinical chemistry analyzer (Mindray BS-200, Shenzhen, China) using commercially available kits (Spinreact, Barcelona, Spain). 

The determination of cardiometabolic risk factors (CRFs) was performed according to the criteria of the National Cholesterol Education Program (NCEP) Expert Panel on Detection, Evaluation, and Treatment of High Blood Cholesterol in Adults [19], considering triglyceride levels ≥ 150 mg/dL, total cholesterol ≥ 200 mg/dL, LDL-C ≥ 100 mg/dL, HDL-C in women < 50 mg/dL and in men < 40 mg/dL. For elevated glucose levels, a cut-off value ≥ 100 mg/dL was considered, according to the International Diabetes Federation [16]. 

### 2.5. Biomarkers of Intestinal Permeability and Inflammation 

Serum zonulin concentrations were measured by enzyme-linked immunosorbent assay with a commercial kit (Human Zonulin ELISA kit, Biomatik, Wilmington, DE, USA) with a sensitivity limit of 0.156 ng/mL and a detection range of 0.625–40 ng/mL. Serum LPS levels were determined with the endotoxin chromogenic quantification kit (Thermo Scientific, Rockford, Illinois, USA), which is an end-point method using an amebocyte lysate with a sensitivity range of 0.1–1.0 EU/mL. High sensitivity C-reactive protein (hsCRP) levels were measured by turbidimetry assay (BioSystems SA, Costa Brava, Barcelona, Spain), the sensitivity was 0.06 mg/L, with intra assay and inter assay variation coefficients of 1.8% and 3.6% respectively. Biomarker levels were considered high above the third tertile: zonulin ≥ 51.6 ng/mL, LPS ≥ 1.35 EU/mL, and hs-CRP ≥ 4.3 mg/L.

### 2.6. Statistical Analysis

Statistical analysis was performed using STATA version 12.0. Quantitative variables with normal distribution are presented as means and standard deviation, quantitative variables without normal distribution as medians and interquartile range, and frequencies for qualitative variables. ANOVA or Kruskal–Wallis tests were used to compare the means or medians of the different variables between groups, and the X^2^ test or Fisher’s exact test was used to compare frequencies. Linear regression models were used to evaluate the interaction between biomarkers of intestinal permeability and inflammation and nutrient intake on metabolic parameters, and these models were adjusted for age, sex and physical activity. Statistical tests were bilateral and a *p*-value <0.05 was considered statistically significant.

## 3. Results

A total of 238 young subjects, of whom 56% were women and 44% were men, with an average age of 20 years were evaluated. To compare the anthropometric and biochemical measurements, we stratified into two groups the study subjects, in the first group were young subjects with 0–2 cardiometabolic risk factors (CRF), and in the second group subjects with 3 or more risk factors. In Table 1, significant differences were observed between both groups in most variables, except for HDL-C levels. In accordance with expectations, the group with ≥3 CRF had higher adiposity and metabolic parameters, as well as more frequent central obesity (83%), hypertrygliceridemia (50%), hypercholesterolemia (68%) and high LDL-C (73%). 

In order to evaluate the nutritional dietary content of the foods consumed by all participants in the study, it was found that the group with ≥3 CRF had a lower intake of PUFA (5 vs. 7 g) and fiber (15 vs. 20 g) compared to the group with <3 CRF (Table 2)

**Figure 1:**
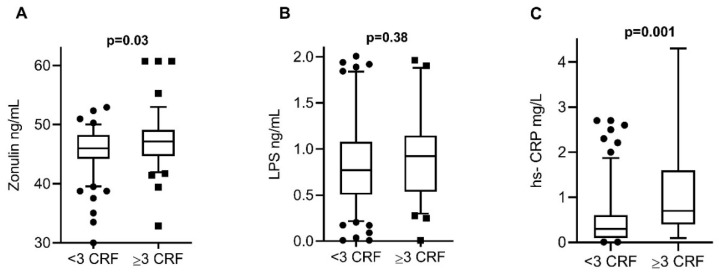
Serum biomarker levels among the study groups. (**A**) Zonulin levels were significantly lower in subjects with <3 CRF in comparison with subjects ≥3 CRF. (**B**) LPS levels were similar in subjects with <3 CRF and in subjects with ≥3 CRF. (**C**) hs-CRP levels were significantly lower in subjects with <3 CRF in comparison with subjects ≥3 CRF. The data are expressed in medians and percentiles p5–p95. *p*-values calculated by the Mann–Whitney U test. *p*-values < 0.05 were considered statistically significant. CRF, cardiovascular risk factors; LPS, lipopolysaccharide; hs-CRP, high-sensitivity C-reactive protein.

To evaluate intestinal permeability and inflammation in both groups, serum concentrations of zonulin, LPS, and hs-CRP were measured, finding elevated concentrations of zonulin and hs-CRP in the group with 3 or more CRF. The levels of the 3 biomarkers analyzed were detected in all serum samples of the study participants. Zonulin levels were significantly lower in subjects with <3 CRF (median 46 ng/mL, 39–50 ng/mL) in comparison with subjects ≥3 CRF (median 48 ng/mL, 43–53 ng/mL, *p* = 0.03) (Figure 1A). LPS levels were similar in subjects with <3 CRF (median 0.77 ng/mL, 0.3–1.8 ng/mL) and in subjects with ≥3 CRF (median 0.92 ng/mL, 0.4–1.9 ng/mL, *p* = 0.38) (Figure 1B). hs-CRP levels were significantly lower in subjects with <3 CRF (median 0.3 mg/L, 0.1–1.9 mg/L) in comparison with subjects ≥3 CRF (median 0.7 mg/L, 0.1–4.2 mg/L, *p* = 0.001) (Figure 1C).

The relationship of nutrient intake with metabolic parameters was determined by linear regressions adjusted for age, gender, and physical activity. Significant associations were found between cholesterol intake and triglyceride levels (β = 3.1, *p* = 0.01); cholesterol levels with total fat (β = 12.4, *p* = 0.04), and SFA intake (β = 2.1, *p* = 0.01); PUFA consumption with HDL-C levels (β = 0.9, *p* = 0.03), and lower triglyceride levels with fiber consumption (β = −16.6, *p* = 0.03).

Regarding the association of each biomarker with metabolic parameters, significant associations were found for LPS with glucose (β = 0.5, *p* = 0.001) and with LDL-C levels ( β = 2.3, *p* = 0.004), and for hs-CRP with triglyceride (β = 0.9, *p* = 0.001) and with total cholesterol levels (β = 2.9, *p* = 0.02, respectively).

Interaction models were performed between biomarkers of intestinal permeability and inflammation to determine their relationship with metabolic parameters. Significant differences were found in the interaction of increased concentrations of zonulin + LPS [β = 1.1 (0.3, 1.9) *p* = 0.006] and LPS + hs-CRP [β = 1.2 (0.3, 3.2) *p* = 0.007] with the increase in glucose levels. A significant increase in triglyceride levels was also observed in the presence of LPS + hs-CRP levels [β = 3.9 (1.2, 8.5) *p* = 0.01]. Furthermore, a trend of LDL-C increased levels was found with the interaction of high levels of zonulin + LPS [β = 3.2 (0.3, 6.8) *p* = 0.07], and LPS + hs-CRP and [β = 3.3 (0.3, 7.8) *p* = 0.08] (Table 3).

Interaction models were performed between high macronutrient intake, elevated levels of zonulin, LPS, or hs-CRP and increased metabolic parameters. It was observed that increased zonulin concentration plus high total fat intake significantly increased total cholesterol levels (β = 8.5, *p* = 0.03). Elevated LPS concentration with high intake of total energy (β = 2.9, *p* = 0.001), carbohydrates (β = 2.9, *p* = 0.001), proteins (β = 2.2, *p* = 0.001), total fat (β = 1.8, *p* = 0.03), and SFA (β = 1.6, *p* = 0.02) significantly increases serum glucose levels. In addition, elevated levels of LPS with high intake of cholesterol or saturated fat, increased triglyceride levels (β = 4.3, *p* = 0.03 and β = 8.1, *p* = 0.04). High hs-CRP levels with the high consumption of carbohydrates (β = 12.3, *p* = 0.02), total fat (β = 5.4, *p* = 0.03) and SFA (β = 9.2, *p* = 0.04), raised triglyceride levels. Similarly, elevated hs-CRP levels with the high intake of total energy (β = 12.5, *p* = 0.006), carbohydrates (β = 11.3, *p* = 0.01), proteins (β = 10.7, *p* = 0.01), and total fat (β = 16.6, *p* = 0.001) increased cholesterol levels. Similarly, elevated hs-CRP levels with high total fat intake (β = 10.7, *p* = 0.01), increased LDL-C levels (Table 4). 

Lastly, interaction models were performed between high macronutrient intake, high levels of zonulin, LPS, or hs-CRP, and the decrease in metabolic parameters. Findings showed that triglyceride levels decreased significantly with higher water intake, even when zonulin (β = −9.6, *p* = 0.03), and LPS (β = −4.8, *p* = 0.02) levels were elevated; likewise, triglyceride levels decreased when there was a higher PUFA intake and high hs-CRP levels (β = −1.3, *p* = 0.01), including higher fiber intake and high hs-CRP levels (β = −1.5, *p* = 0.02). Total cholesterol concentrations decreased when higher PUFA was consumed, even when hs-CRP levels were increased (β = −5.1, *p* = 0.01). LDL-C levels also decreased when more fiber was consumed, although LPS levels were elevated (β = −7.7, *p* = 0.01) (Table 5). 

## 4. Discussion

In this study, we found that 35% of the young adults presented a metabolic profile of cardiovascular risk with the most frequent alterations being low HDL-C levels, high LDL-C levels, and central obesity. In addition, increased intestinal permeability and low-grade inflammation, determined by high levels of zonulin and hs-CRP, were observed in the study group with ≥3 CRF. Findings also showed that an increased intestinal permeability and the presence of low-grade inflammation with a high- carbohydrate and total fat diet favored the increase of glucose, triglyceride, and total cholesterol levels. It is important to mention that the increased consumption of water, fiber, and PUFA can reduce serum triglyceride, total cholesterol, and LDL-C levels, despite increased levels of zonulin, LPS, or hs-CRP.

In Mexico, according to the 2016 National Health and Nutrition Survey for the population aged 20–29 years, the prevalence of central obesity for women was 75.3% and 51% in men, unlike this study, where men showed a higher prevalence compared to women (52% vs. 35%) [20]. With respect to the lipid profile, 13.9% of hypercholesterolemia was reported, and in this study, it was 31% and hypertriglyceridemia 18%. Abdominal obesity increases the risk of cardiovascular disease (CVD) and type 2 diabetes mellitus (T2DM) because visceral adipocytes in response to excess lipid reserves secrete more inflammatory cytokines, such as tumor necrosis factor alpha (TNF-α), interleukin 6 (IL-6), and chemokines like monocyte chemoattractant protein 1 (MCP-1). Chemokines, in turn, promote macrophage migration to the adipose tissue increasing cytokine release and leading to a systemic inflammatory state, which is associated with insulin resistance and altered lipid profile [21].

In this study, an increase in serum levels of zonulin and hs-CRP was observed in young subjects with ≥3 CRF. These proteins were determined as markers of intestinal permeability and low-grade inflammation, suggesting that under these conditions permeability dysfunction and a proinflammatory state may occur. Fasano et al. discovered and characterized zonulin as the only protein that can reversibly modulate intestinal permeability and studies indicate that it can alter permeability in obesity. In patients with excess weight, functional tests have shown an increase in intestinal permeability, finding an increased relationship between the ingestion of carbohydrates, such as mannitol and lactulose and their urinary excretion, suggesting an increase in both absorption and excretion [22,23]. Associations have also been found between increased serum zonulin levels with increased BMI, waist-to-hip ratio, and triglyceride levels, as well as with high fasting insulin levels; thus, it has been proposed that increased zonulin may be related to insulin resistance [24].

Increased serum zonulin levels suggest dysfunction of the intestinal barrier, allowing the entry of toxins from the lumen into the blood circulation. One of these endotoxins is LPS, which is released from bacterial lysis as it forms a structural part of the cell walls of Gram-negative bacteria. Increased endotoxins can trigger local inflammation or access the circulation and induce systemic inflammation through cytokine release [25]. Increased expression and activation of CRP and proinflammatory cytokines, such as IL-6, are associated with atherogenesis and CVD. Therefore, inflammatory biomarkers are considered predictors of future cardiovascular events, however, hs-CRP is a stronger and independent predictor of CVD than other inflammatory markers and even serum lipids [26].

The Western diet has also been found to be highly associated with the risk of developing metabolic syndrome [27]. In this study, a daily cholesterol intake >250 mg was found to increase triglyceride levels. Other studies have found that a high cholesterol intake influences an increase in both LDL-C and HDL-C. The relationship between cholesterol intake and triglyceride levels can be attributed to the fact that most animal foods that are high in cholesterol are also rich in saturated fatty acids, thus favoring an increase in serum triglycerides [28]. An association was also found between SFA (>24.6 g), increased total cholesterol and LDL-C concentrations. This effect may be attributed to the fact that SFA increased cholesterol levels and decreased LDL receptor activity, as well as the amount of protein and mRNA, whereas unsaturated fatty acids have the opposite effect [29]. Lin et al. demonstrated that SFA intake induces the expression of peroxisome proliferator-activated receptor gamma co-activator 1 (PGC-1) and enzymes involved in cholesterol biosynthesis, such as 3-hydroxy-3-methylglutaryl coenzyme A reductase (HMG-CoA reductase), phosphomevalonate kinase, and lanosterol synthase, among others, causing an increase in LDL cholesterol [30].

Our results show that higher consumption of PUFA contributes to decreasing triglyceride and cholesterol levels, just as the high consumption of fiber decreases triglyceride levels. Contrary to our results, another study reported reduced total cholesterol and HDL-C levels when foods rich in saturated fats were replaced with foods rich in n-6 PUFA, [31]. In regard to the consumption of dietary fiber, it has been shown to decrease postprandial triglyceride and cholesterol concentrations due to slower gastric emptying, alteration of triglyceride hydrolysis, through increased viscosity that decreases the rate of hydrolysis or inhibition of pancreatic lipase activity, which is consistent with our results [32].

Biomarkers of intestinal permeability and inflammation were analyzed in an integrated manner with nutrient intake and their effect on cardiometabolic risk factors and findings showed that the increase in intestinal permeability, determined by the increase in serum zonulin, together with high total fat intake, significantly increased cholesterol levels. In a study by Mörkl et al., women with higher zonulin levels had higher intakes of total calories, protein, and fat; similarly, Zak-Golab et al. found a correlation between serum zonulin and a higher fat intake. Both studies proposed that increased intestinal permeability favors fat absorption, increasing serum cholesterol and triglyceride concentrations [33,34]. This work showed that increased circulating LPS plus a high intake of total calories and macronutrients significantly increase glucose and triglyceride levels. Mani et al. demonstrated that LPS concentration increases with a meal rich in SFA, while it decreases after a meal rich in n-3 PUFA. This effect was attributed to the fact that some SFA, such as lauric and myristic acid are part of lipid A, which is a component of LPS and could favor the activation of Toll-like receptors (TLRs). Several studies have shown that the consumption of an SFA-rich diet over a prolonged period results in higher numbers of Gram-negative bacterial populations, while diets high in fiber increase Gram-positive bacterial populations. One of the proposed mechanisms by in vitro studies is that fatty acids directly activate TLR-4 and TLR-2 signaling pathways. This finding has led to the hypothesis that TLRs are a molecular link between diet and lipids, inflammation, and innate immune cell activation in the regulation of insulin sensitivity in response to dietary changes [35,36,37]. Saturated fatty acids whose levels are significantly increased in plasma and adipose tissue of obese individuals act endogenously by activating TLR4. Recently, it has been proposed that obesity-related changes in the composition of the gut microbiota may favor increased intestinal permeability and the entry of LPS into the blood circulation [38]. Therefore, LPS and SFA could act together to induce TLR4 dimerization and activation and thus, promote inflammation in obesity.

Furthermore, in this study increased serum levels of hs-CRP and a high intake of calories, carbohydrates, proteins, and fats increased triglyceride, total cholesterol, and LDL-C concentrations. In other studies, it has been reported that metabolic syndrome and its components are associated with inflammatory status and subjects with metabolic syndrome show a high concentration of hs-CRP [39]. C-reactive protein (CRP) is produced in response to stimulation by interleukins, such as IL-6. A Western diet is rich in red meat, high-fat dairy products, refined grains, and simple carbohydrates, and it has been associated with higher levels of CRP and IL-6 and thus can induce chronic inflammation [40].

In relation to carbohydrate consumption and low-grade inflammation, it has already been determined that the high glycemic index of certain foods is associated with increased markers of inflammation rather than the amount of carbohydrate ingested. Kallio et al. found that the long-term consumption of cereals affects postprandial insulin secretion and inflammatory status. They mention that a diet rich in whole grains does not produce inflammation, whereas a high-glycemic index diet produces low-grade inflammation [41]. Regarding the relationship between increased hs-CRP levels with high protein intake and metabolic risk factors, it can be attributed to the fact that consumption is not differentiated between animal and vegetable protein, since red meat is known to have a high SFA content, and as a result, the inflammatory gene expression is directly stimulated through TLR4 signaling [42]. Furthermore, processing meats at high temperatures originates advanced glycation end products (AGEs) that are associated with oxidative stress and induce nuclear factor kB (NF-kB) activation and proinflammatory cytokine synthesis [43].

Interestingly, it was observed that high intakes of fiber, water, and PUFA had an effect on the reduction of serum concentrations of triglycerides, total cholesterol and LDL-C, despite an increase in biomarkers of permeability and low-grade inflammation. In different studies, it has been observed that a healthy dietary pattern, such as frequent consumption of fiber, including monounsaturated and polyunsaturated fats, can improve insulin sensitivity, increase antioxidant defense, and thus reduce the risk of metabolic disorders [44]. Other studies have also found that CRP levels decrease with diets rich in fruits and vegetables, as well as with the consumption of PUFA and fiber [45,46,47].

It has also been documented that the consumption of fiber can lower serum glucose and lipid levels. The lowering effect of fiber on cholesterol levels may be attributed to the increased excretion of fecal bile salts, while the effect on triglyceride levels is attributed to delayed and reduced absorption of these lipids and sugars in the small intestine [48]. Furthermore, high fiber intake could also modify the intestinal microbiota and increase the abundance of short-chain fatty acid (SCFA)-producing bacteria, which are related to the integrity of the intestinal wall by improving permeability [49].

## 5. Conclusions

The results of this study suggest that higher consumption of fats and carbohydrates in the presence of intestinal permeability dysfunction and low-grade inflammation increases serum glucose and lipid levels, but when there is a higher intake of water, PUFA and fiber, glycemic and lipid profiles are improved. Nonetheless, these results should be validated in a controlled clinical study or in longitudinal studies in which long-term dietary patterns are evaluated to confirm the beneficial effect on the cardiometabolic profile.

## Figures and Tables

**Table 1:** Anthropometric and biochemical characteristics of young subjects according to cardiovascular risk factors.

Variables	Total *n* = 238	<3 CRF *n* = 154 (65)	≥3 CRF *n* = 84 (35)	*p* Value
**Sex *n* (%)**				<0.001 ^a^
Women	134 (56)	98 (64)	36 (43)	
Men	104 (44)	56 (36)	48 (57)	
**Age (years)**	20 (18–22)	19 (18–21)	21 (19–24)	0.009 ^c^
**Weight (Kg)**	64.6 (53–80)	58 (50–67)	80.5 (71–88)	<0.001 ^c^
**BMI (Kg/m^2^)**	25 (20.8–29)	21.9 (20–25.5)	30 (27–33)	<0.001 ^c^
**WC (cm)**				
Women	75 (68–83)	72 (66–77)	87 (81–95)	<0.001 ^c^
Men	91 (77–100)	78 (71–85)	98 (93.5–103)	<0.001 ^c^
**HC (cm)**	98 (91–100)	93 (87–101)	107 (103–112.5)	<0.001 ^c^
**WtHR**	0.8 (0.76–0.89)	0.78 (0.74–0.82)	0.89 (0.83–0.93)	<0.001 ^c^
**BP (mmHg)**				
Systolic	108 (100–116)	105 (97–112)	113 (107–125)	<0.001 ^c^
Diastolic	65 (59–73)	62 (58–70)	71 (64–67)	<0.001 ^c^
**Fat mass (Kg)**	18.6 (11.9–25)	14.2 (10–20.3)	25 (20–29.8)	<0.001 ^c^
**Muscle mass (Kg)**	43.7 (37.7–54.7)	40.6 (36.3–48.6)	54 (44.6–61)	<0.001 ^c^
***Metabolic parameters***				
**Glucose (mg/dL)**	79 (73–85)	78 (72–85)	81 (74–89)	0.003 ^c^
**TG (mg/dL)**	85 (63–135)	72 (58–95)	152 (101–182)	<0.001 ^c^
**TC (mg/dL)**	179 (148–213)	165 (138–188)	216 (181–244)	<0.001 ^c^
**LDL-C (mg/dL)**	101 (79–121)	90 (72–107)	148 (117–180)	<0.001 ^c^
**HDL-C (mg/dL)**				
Women	40 (37–42)	40 (37–42)	40 (38–41)	0.470 ^c^
Men	40 (37–42)	41 (37–43)	38 (37–40)	0.200
***Cardiometabolic risk factors n (%)***				
**Central obesity**	98(41)	28(18)	70(83)	<0.001 ^a^
Women (≥80 cm)	47(35)	17(17)	30(83)	<0.001 ^a^
Men (≥90 cm)	51(49)	11(20)	40(83)	<0.001 ^a^
**High blood pressure**	17(7)	1(1)	16(19)	<0.001 ^b^
**Glucose ≥ 100 mg/dL**	7(3)	4(3)	3(4)	0.48 ^b^
**TG ≥ 150 mg/dL**	44(18)	2(1)	42(50)	<0.001 ^b^
**TC ≥ 200 mg/dL**	74(31)	17(11)	57(68)	<0.001 ^a^
**LDL-C ≥ 100 mg/dL**	102(43)	41(27)	61(73)	<0.001 ^a^
**Low HDL-C**	172(72)	108(70)	64(76)	0.32 ^a^
Women (<50 mg/dL)	124(93)	91(93)	33(92)	0.53 ^b^
Men (<40 mg/dL)	48(46)	17(30)	31(65)	<0.001 ^a^

Data represented as frequency and percentage *n* (%) or median (25th–75th percentiles). *p*-values were calculated by ^a^ Chi-square test, ^b^ Fisher’s exact test or ^c^ Mann–Whitney U test. A *p*-value < 0.05 was considered statistically significant. CRF, cardiovascular risk factors; BMI, body mass index; WC, waist circumference; HC, hip circumference; WtHR, waist-to-hip ratio; BP, blood pressure; TG, triglycerides; TC, total cholesterol; LDL-C, low density lipoprotein-cholesterol; HDL-C, high density lipoprotein-cholesterol.

**Table 2:** Intake of energy and nutrients according to cardiovascular risk factors.

Nutrient	Total *n* = 238	<3 CRF *n* = 154 (65)	≥3 CRF *n* = 84 (35)	*p* Value
Energy (cal)	1816 (1298–2459)	1809 (1342–2382)	1861 (1184–2991)	0.66
CHO (g)	226 (157–337)	213 (154–305)	253 (161–387)	0.12
Protein (g)	73 (51–101)	75 (52–100)	66 (46–108)	0.53
Lipid (g)	66 (39–98)	64 (42–94)	77 (38–110)	0.69
SFA (g)	13 (7–19)	12 (7–19)	15 (8–19)	0.46
PUFA (g)	6 (3–8)	7 (4–8)	5 (3–7)	0.007
Cholesterol (mg)	129 (66–273)	127 (73–273)	133.5 (57–223)	0.75
Water (mL)	1167 (815–1765)	1196 (824–1816)	1141 (727–1597)	0.37
Fiber (g)	18 (12–25)	20 (13–25)	15 (9–24)	0.03

Data represented as median (25th–75th percentiles). *p*-values were calculated by Mann–Whitney U test. A *p*-value < 0.05 was considered statistically significant. Energy, total energy; CHO, carbohydrates; SFA, saturated fatty acids; PUFA, polyunsaturated fatty acids.

**Table 3:** Interaction between intestinal permeability and inflammation biomarkers on cardiometabolic risks factors in all participants.

	Glucose β (95% CI) *p*	Triglycerides β (95% CI) *p*	Cholesterol β (95% CI) *p*	LDL-C β (95% CI) *p*	HDL-C β (95% CI) *p*
↑Zonulina + ↑LPS	1.1 (0.3, 1.9) 0.006	2 (−0.3, 2.5) 0.3	2.3 (0.7, 6) 0.2	3.2 (0.3, 6.8) 0.07	0.01 (−0.33, 0.3) 0.9
↑Zonulina + ↑hs-CRP	0.6 (−0.9, 2.1) 0.4	2.2 (0.03, 7) 0.4	0.8 (−3, 5) 0.6	1 (0.4, 4) 0.5	−0.2 (−0.6, 0.3) 0.1
↑LPS + ↑hs-CRP	1.2 (0.3, 2) 0.007	3.9 (1.2, 8.5) 0.01	3.8 (0.7, 8) 0.1	3.3 (0.3, 7.8) 0.08	−0.1 (−0.5, 0.2) 0.4

Regression model adjusted for age, sex and physical activity. Data are presented in β-coefficient and 95% confidence Interval. *p*-value < 0.05 was considered statistically significant. High levels (↑) of biomarkers were considered above the third tertile (Zonulin ≥ 51.6 ng/mL, LPS ≥ 1.35 EU/mL, hs-CRP ≥ 4.3 mg/L). LPS, lipopolysaccharide; hs-CRP, high-sensitivity C-reactive protein; LDL-C, low density lipoprotein-cholesterol.

**Table 4:** Interaction between nutrient intake and biomarkers of intestinal permeability and inflammation on the increase of metabolic parameters in all participants.

	Glucose β (95% CI) *p*	Triglycerides β (95% CI) *p*	Cholesterol β (95% CI) *p*	LDL-C β (95% CI) *p*	HDL-C β (95% CI) *p*
↑Zonulin + ↑Energy	1.1 (−1.9, 4) 0.4	4.8 (0.3, 5.7) 0.3	0.5 (−7, 8.8) 0.9	3.5 (0.5, 10) 0.3	0.3 (0.1, 1) 0.3
↑Zonulin + ↑CHO	1.7 (−1.3, 4.7) 0.2	3 (0.4, 7) 0.5	0.7 (−7, 9.8) 0.8	1.1 (0.3, 6) 0.7	0.1 (0.01, 0.6) 0.7
↑Zonulin + ↑Protein	2 (−1.1, 5.3) 0.2	4.2 (0.6, 15.2) 0.4	4 (0.5, 12) 0.3	1.4 (−5, 8) 0.6	0.2 (0.04, 0.9) 0.5
↑Zonulin + ↑Fat	0.1 (−2.8, 3.2) 0.9	−0.4 (−10.6, 9) 0.9	8.5 (0.5, 16) 0.03	0.2 (−7, 7) 0.9	0.2 (−0.6, 1.0) 0.6
↑Zonulin + ↑Chol	−1.6 (−4.9, 1.6) 0.3	3 (0.2, 8.1) 0.6	0.4 (0.2, 0.6) 0.9	2.5 (−4.9, 10) 0.5	0.7 (0.05, 1.5) 0.06
↑Zonulin + ↑SFA	0.3 (−2.3, 3) 0.8	9.2 (0.2, 18) 0.4	5.3 (0.6, 12) 0.1	−0.5 (−6, 5) 0.8	0.6 (00.02, 1.2) 0.06
↑LPS + ↑Energy	2.93 (1.3, 4.7) 0.001	1 (0.1, 10.7) 0.8	0.7 (−9, 8.3) 0.9	1.6 (−9, 5) 0.6	0.5 (0.2, 1.2) 0.2
↑LPS + ↑CHO	2.9 (1.2, 4.5) 0.001	3.5 (−5, 12.7) 0.5	1.6 (−10, 7.8) 0.7	0.8 (−6.3, 8) 0.8	0.1 (0.01, 0.5) 0.6
↑LPS + ↑Protein	2.2 (0.6, 3.8) 0.001	−0.6 (−9.9, −1.0) 0.08	1.5 (−10.5, 7) 0.7	0.2 (−7, 7) 0.9	0.7 (0.04, 0.9) 0.07
↑LPS + ↑Fat	1.8 (0.1, 3.5) 0.03	0.8 (−8.9, 10.6) 0.9	6.3 (−0.3, 15) 0.1	6.3 (−1.3, 14) 0.1	0.7 (−0.01, 1.4) 0.05
↑LPS + ↑Chol	0.3 (−1.3, 1.9) 0.7	4.3 (0.6, 13.1) 0.03	2.3 (0.1, 6) 0.6	4.3 (−2.8, 11) 0.2	0.4 (−0.2, 1.1) 0.1
↑LPS + ↑SFA	1.6 (0.1, 3) 0.02	8.1 (0.18, 16) 0.04	0.2 (−7.6, 7) 0.9	2.3 (−4, 8) 0.5	0.6 (0.01, 1.2) 0.05
↑hs-CRP + ↑Energy	0.1 (−3.2, 3.4) 0.9	9.9 (1.2, 21.2) 0.08	12.5 (3, 21) 0.006	3.3 (0.5, 11) 0.4	0.4 (0.1, 2) 0.4
↑hs-CRP + ↑CHO	0.3 (−2.9, 3.6) 0.8	12.3 (1.4, 23) 0.02	11.3 (2.7, 19.8) 0.01	0.7 (−8.1, 6.6) 0.8	−0.03 (−0.1, 0.4) 0.3
↑hs-CRP + ↑Protein	4.2 (0.9, 7.5) 0.01	1.7 (−1.4, 20.2) 0.8	10.7 (1.9, 19) 0.01	1.9 (−9, 5) 0.6	0.1 (−0.06, 0.9) 0.7
↑hs-CRP + ↑Fat	0.7 (−4.1, 2.6) 0.7	5.4 (1.6, 16) 0.03	16.6 (7.8, 25) 0.001	10.7 (1.9, 7) 0.01	0.2 (−0.6, 1.0) 0.6
↑hs-CRP + ↑Chol	−2.7 (−4.8, 0.4) 0.09	3.9 (−0.6, 12.1) 0.4	6.6 (0.2, 15.6) 0.04	3.1 (−10.9, 3) 0.4	0.2 (−0.5, 0.6) 0.5
↑hs-CRP + ↑SFA	1.2 (−1.3, 9) 0.4	9.2 (0.2, 18) 0.04	4.8 (0.6, 12) 0.1	3.5 (−6, 5) 0.3	−0.6 (−1.2, 0.2) 0.06

Regression model adjusted for age, sex, and physical activity. Data are presented in β coefficient and 95% confidence Interval. *p*-value < 0.05 was considered statistically significant. High levels (↑) of biomarkers (Zonulin ≥ 51.6 ng/mL, LPS ≥ 1.35 EU/mL, hs-CRP ≥ 4.3 mg/L) and high intake (↑) of nutrients (Total fat ≥ 138 g, Total energy ≥ 3236 kcal, CHO ≥ 406 g, Protein ≥ 133 g, Chol ≥ 445 mg, ≥SFA 24.6 g), were considered above the third tertile. CHO, carbohydrate; Chol, cholesterol; SFA, saturated fatty acids; LPS, lipopolysaccharide; hs-CRP, high-sensitivity C-reactive protein; LDL-C, low density lipoprotein-cholesterol; HDL, high density lipoprotein-cholesterol.

**Table 5:** Interaction between nutrient intake and biomarkers of intestinal permeability and inflammation on the decrease of metabolic parameters in all participants.

	Glucose β (95% CI) *p*	Triglycerides β (95% CI) *p*	Cholesterol β (95% CI) *p*	LDL-C β (95% CI) *p*	HDL-C β (95% CI) *p*
↑Zonulina + ↑PUFA	−0.4 (−3, 2.1) 0.7	−6.8 (−15.8, −2.1) 0.1	−0.4 (−0.7, −0–1) 0.09	−0.5 (−6.5, 5) 0.9	0.5 (0.1, 0.6) 0.8
↑Zonulina +↑Fiber	−0.7 (−3.4, 1.9) 0.6	−5.7 (−14.7, −3.3) 0.2	1.2 (−5.8, 8.1) 0.7	−4.5 (−10, −1.5) 0.1	0.8 (0.01, 6) 0.9
↑Zonulina + ↑Water	−0.3 (−3, 2.3) 0.8	−9.6 (−18.65, −0.6) 0.03	−4.2 (−11.2, 0.2) 0.2	−0.5 (−6.5, 5) 0.8	0.8 (0.2, 1) 0.7
↑LPS + ↑PUFA	0.8 (−5, 2.2) 0.2	−0.9 (−8.8, −0.1) 0.1	−0.5 (−7, −8) 0.9	−4.5 (−6, 11.5) 0.1	0.09 (0.001, 0.5) 0.8
↑LPS + ↑Fiber	−0.9 (−0.5, 2.2) 0.2	−2.79 (−10.9, −0.3) 0.4	2.8 (−4.8, 10) 0.5	−7.7 (−13, −1.4) 0.01	0.2 (−0.4, 0.8) 0.5
↑LPS + ↑Water	−1.3 (−3, 2.3) 0.06	−4.8 (−12.6, −3.6) 0.02	−3.3 (−10.2, 0.4) 0.4	−5.7 (−11.5, 0.5) 0.06	0.17 (−0.4, 0.7) 0.6
↑hs-CRP + ↑PUFA	−1.0 (−3.7, 1.6) 0.4	−1.3 (−7.8, −0.1) 0.01	−5.1 (−7, −0–1) 0.01	−0.3 (−6.3, 5.7) 0.9	−0.57 (−1.1, 0.3) 0.06
↑hs-CRP + ↑Fiber	−1.6 (−4.3, 1) 0.2	−1.5 (−10.5, −0.3) 0.02	4.3 (−2.7, 8.1) 0.2	−0.3 (−2, 6.5) 0.2	−0.4 (−0.8, 0.2) 0.1
↑hs-CRP + ↑Water	−1.5 (−4.2, 1.2) 0.2	−0.8 (−8.3, 10.6) 0.8	−2 (−5, 0.9) 0.6	−3.7 (−9.9, 2) 0.2	−0.5 (−1.2, 1) 0.1

Regression model adjusted for age, sex and physical activity. Data are presented in β coefficient and 95% confidence interval. *p*-value < 0.05 was considered statistically significant. High levels (↑) of biomarkers (Zonulin ≥ 51.6 ng/mL, LPS ≥ 1.35 EU/mL, hs-CRP ≥ 4.3 mg/L) and high intake (↑) of nutrients (Water ≥ 2100 mL, Fiber ≥ 30 g, PUFA ≥ 6 g), were considered above the third tertile. PUFA, polyunsaturated fatty acids; LPS, lipopolysaccharide; hs-CRP, high-sensitivity C-reactive protein; LDL-C, low density lipoprotein-cholesterol; HDL, high density lipoprotein-cholesterol.

## Data Availability

The data used to support the findings of this study is available from the corresponding authors upon reasonable request.
